# Harnessing the microbiome to improve clinical outcomes for cancer, transplant, and immunocompromised patients in the intensive care unit (ICU)

**DOI:** 10.3389/fcimb.2025.1577108

**Published:** 2025-06-12

**Authors:** Lizbeth Nieves, Alexandra Roach, Joseph Hunter, Sarah Smeh, Andrew Islas, Ariana Islas, Joseph Blattman, Michelle Di Palma

**Affiliations:** ^1^ School of Life Sciences, Arizona State University, Tempe, AZ, United States; ^2^ Department of Medicine, University of Arizona College of Medicine, Phoenix, AZ, United States

**Keywords:** microbiome, dysbiosis, immunocompromised, cancer, transplant, pathobiome, intensive care unit, ICU

## Abstract

In recent decades, there has been a growing emphasis on understanding how the architecture of the human microbiome can impact typical biological processes and patient clinical outcomes. In fact, microbiome modifications and modulations have not only been associated with impacts on general health and well-being but have also been shown to yield differences in patient responsiveness to vaccines, medications, and chemotherapeutic regimens. Much of this influence likely stems from how changes in the microbiome result in differences in microbial communities and the subsequent release of microbial-derived metabolites that can alter typical immunological processes. Understanding how microbial composition can impact patient responsiveness can be particularly important in the intensive care unit (ICU), where the efficacy of medications and treatments can result in negative patient outcomes if unsuccessful. Clinical scientists have further developed the concept of the pathobiome, a disease-promoting microbiome whose development can be associated with dysbiosis. Understanding how the microbiome and its associated components can impact patient responsiveness, especially in the ICU, must be further researched and understood. Here, we analyze what causes variances in the microbiome and pathobiome in significant immunocompromised populations, including cancer patients and transplant recipients, and how variances in the microbiome can impact patient outcomes in the ICU. Further, we detail potential future applications of how our understanding of what impacts the human microbiome during the treatment of these populations may be exploited to improve patient prognosis.

## Introduction

The human microbiome is a collective of various microorganisms, including bacteria, fungi, viruses, and their associated byproducts. In a healthy microbiome, thousands of microorganisms interact to create a balanced and resilient ecosystem in both mucosal and epithelial regions. Recently, it has been found that the architecture of the microbiome can strongly impact host biological processes, with many of these impacts being linked not only to general human health but also to disease outcomes (Moloney et al., 2014; Manos, 2022). Thus, an emphasis on how microbiome modulation can impact human health and disease pathology has become of great interest to researchers and medical professionals. Modern advancements such as high-throughput genomic sequencing and metagenomic studies allow researchers to not only dissect the composition of the microbiome but also deepen their understanding of how the products from microbial species impact cellular and organ functions (Shi et al., 2022). Leveraging many of these advancements, the Human Microbiome Project revealed that healthy individuals have substantial diversity within their microbiome populations, highlighting the complexity of understanding its full impact (Huttenhower et al., 2012). Further studies indicate that products made by both the microbiome of the gut and of specific organs can modulate the function of both innate and adaptive immune cells and their subsequent impact on host organs and diseases (Russo et al., 2016; Thaiss et al., 2016). Disruptions to this system, particularly microbial depletion or loss of commensal diversity, can be especially detrimental in immunocompromised and cancer patients, whose immune systems are already burdened. Several studies have indicated that specific microbial compositions can impact disease severity, progression, and responsiveness to treatment for immunodeficient patients (Moloney et al., 2014).

Extreme dysbiosis is characterized by a significant loss of commensal microbes and a dominance of opportunistic pathogens, leading to an imbalance in gut bacteria. This can significantly impact critical illness, increasing susceptibility to nosocomial infection and organ failure (McDonald et al., 2016). Further insight into the formation of the pathobiome, a microbiome characterized by an overabundance of pathogenic microorganisms that advance disease, could deepen our knowledge of how Intensive Care Unit (ICU) patients are impacted by treatment (Munley et al., 2023). Broadening our understanding of the impact of microbial composition on immunodeficiency is essential if we hope to improve treatment strategies for immunocompromised patients. Here, we elucidate how the microbiome’s composition can impact patient disease progression in the context of immunocompromised and cancer patients and disease outcomes for these patients in the ICU. Further, we explain findings from recent studies that have sought to determine potential modulations of the microbiome for patients living with compromised immune systems, with the future potential to exploit these findings to improve patient outcomes in the ICU.

## How microbiome composition influences treatment outcomes in the ICU

Understanding how treatment outcomes for patients in the ICU can be impacted by the presence or absence of certain microbes is vital if clinicians hope to one day tailor treatments to patients to catalyze the best possible outcomes on an individual basis. Here, we detail known associations of specific microbes and microbial compositions with clinical and treatment outcomes in ICU patients.

### General patients in the ICU

**Figure 1:**
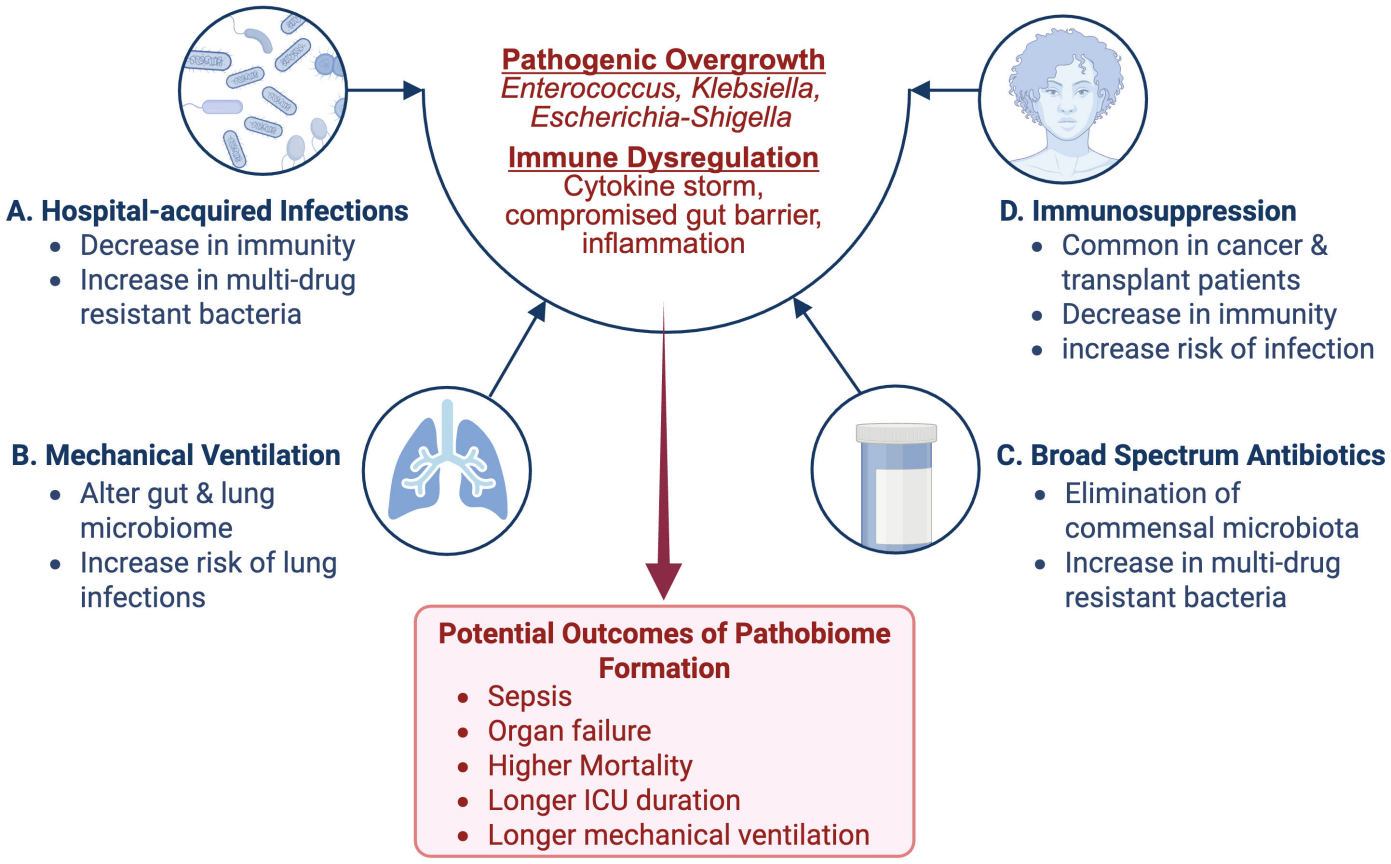
Contributing Factors to Microbiome Dysbiosis and Pathobiome Development in the ICU: Several factors specific to the ICU contribute to microbiome dysbiosis and subsequent pathobiome development. Among these factors are **(A)** the use of broad-spectrum antibiotics (ATB) can kill off commensal microbiota, allowing for resistant and potentially pathogenic microbiota to flourish; **(B)** the use of mechanical ventilation, which can alter the mucosal microbiome and increase the risk for lung infections; **(C)** Immunosuppression, which is common in the case of cancer and transplant patients, can lead to a decrease in immune defenses and increased infection risk **(D)** Hospital-acquired infections (HAIs), which can weaken immune defenses and introduce multidrug-resistant bacteria. Image created with BioRender.com.

An estimated 5.7 million patients are admitted annually to ICU in the United States, with this number expected to grow due to the aging population and subsequent increasing chronic health conditions (Viglianti and Iwashyna, 2017). Within this population, the microbiome can be depleted to as few as four main species, which compete for dominance, heightening susceptibility to hospital-acquired infections (HAIs), multiorgan dysfunction, and ultimately poorer clinical outcomes (Miniet et al., 2021). Globally, alpha-diversity - the measure of number and spread of species within the microbiome - was slightly reduced in early ICU admission of adults and children. While this early measure was not associated with in-hospital mortality, the alpha-diversity was found to decline in ICU patients overtime (Evans et al., 2023). In addition to microbiome depletion, characterized by a reduction in microbial diversity and/or loss of commensal microbes, early evidence suggests that healthy hospital workers do not show increased colonization rates by bacteria associated with HAIs, implying that these organisms and a depleted and/or overtaxed immune system are necessary to create a pathological environment (**Figure 1A**). ICU patients have a higher susceptibility to HAIs due to interrelated factors: microbiome depletion from broad-spectrum antibiotics, immune dysfunction, and frequent barrier compromise from essential medical devices (Wohrley et al., 2018). Proposed mechanisms for this change include decreasing immunological barriers within the gut mucosa. This would include compromised mucosal epithelia, reduced secretory IgA, and impaired immune cell function, which normally would prevent the inward migration of pathogenic species and slow the removal of bacterial colonies from the gut. This dysregulation can heighten the host immune system’s infection risk or inflammatory response, leading to organ system damage. A multifaceted approach is needed to analyze the various microbiota-related factors that contribute to HAIs to both prevent and manage them (Tozzo et al., 2022). For example, patients diagnosed with a *Clostridium difficile* infection are 70% more likely to be re-hospitalized with sepsis, highlighting the importance of minimizing dysbiosis in ICU patients (Nakov et al., 2020). These microbiome-related vulnerabilities are especially crucial in cancer patients, who face not only higher rates of ICU admission but often experience poorer outcomes following intensive care unit interventions (Nazer et al., 2022).

### Cancer patients in the ICU

Recent studies indicate that 5.2-6.4% of cancer patients will develop a critical illness that will result in an ICU visit within two to five years of diagnosis (Bos et al., 2015; Puxty et al., 2015). Lung and colorectal cancer rank as the most fatal, with lung and bronchus cancer accounting for 21% of mortalities (Siegel et al., 2023, 2024). Several studies report that lung cancer has the highest ICU mortality rate and poorest survival rate post-ICU admission, with an average ICU mortality rate of 40.1%. Notably, invasive mechanical ventilation, which is required for up to half of all ICU cancer patients, was a key factor linked to higher mortality (Soares et al., 2007; Andréjak et al., 2011; Puxty et al., 2014) (**Figure 1B**). Further, research suggests a link between microbial diversity and ICU outcomes, particularly during mechanical ventilation.

**Figure 2:**
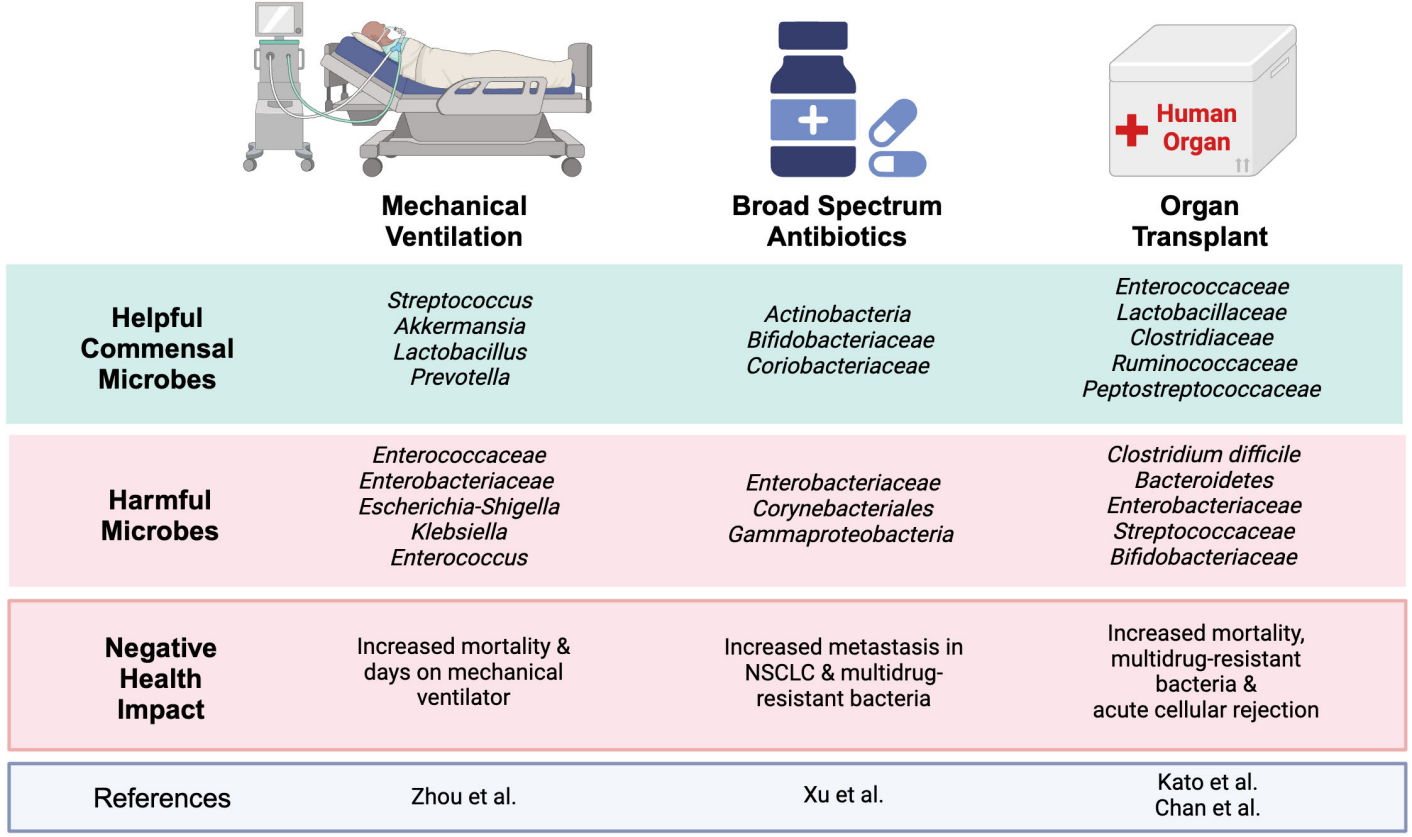
Helpful and harmful microbiota associated with treatment outcomes: A simplified overview of varying studies on ICU interventions and the microbes associated with helpful and harmful clinical outcomes. The gut microbiome is a complex network that has been shown to have drastic impacts on different treatments. Within the ICU, an increase in pathogenic and decrease in commensal bacteria can lead to increased mortality, increased metastasis in patients with NSCLC, and even acute cellular rejection. Image created with BioRender.com.

Recent studies have shown critically ill patients who underwent mechanical ventilation and didn’t survive had significantly lower microbial α-diversity than survivors in their lung and gut, with noted migration of gut microbes to the lungs (Zhou et al., 2023). The diseased group also exhibited a significantly reduced concentration of fecal short-chain fatty acids (SFCAs): pentanoic acid, butyric acid, isobutyric acid, and isovaleric acid (Zhou et al., 2023). Along with a significantly increased amount of *Enterococcaceae* and *Enterobacteriaceae* within the gut and correlated with a 28-day mortality rate (Zhou et al., 2023). Additionally, 44% (27 patients) of the cohort died within 28 days and had significantly lower microbial diversity in both their lungs and gut (P<0.05) compared to the survivors (Zhou et al., 2023) (**Figure 2**). The microbiome of the survival group was enriched with commensal bacteria: *Streptococcus, Akkermansia, Lactobacillus*, and *Prevotella.* The deceased group showed decreased commensal bacteria and increased opportunistic bacteria: *Escherichia-Shigella, Klebsiella*, and *Enterococcus* (**Figure 2**). Further, patients with low lung α-diversity showed significantly higher mortality than those with high lung α-diversity (P<0.01) (Zhou et al., 2023). While this study provides key insights, note that it was completed in a small cohort and at a single center, highlighting the potentially limited application of these findings. Considering the significant link between microbial imbalances, respiratory infections, and poor outcomes in lung cancer patients on mechanical ventilation, similar investigations may be warranted in lung cancer patients with acute respiratory failure (ARF) (Yoo et al., 2013). These findings underscore the growing interest in how microbiome composition influences both respiratory outcomes and treatment efficacy in lung cancer patients.

Although immune checkpoint inhibitors (ICIs) have revolutionized the therapeutic landscape in cancers such as advanced non-small cell lung cancer (NSCLC), the microbiome has been shown to impact their efficacy. A recent study indicated that broad-spectrum antibiotics (ATB) significantly increased metastasis in ATB-treated ARF patients with NSCLC compared to non-ATB treated (P<0.01). These ATB-treated patients had a significant reduction in α-diversity within the gut microbiome, with notable shifts in phyla microbial diversity. In the non-ATB group, the microbiome was enriched with *Actinobacteria*, *Bifidobacteriaceae*, and *Coriobacteriaceae*. In contrast, the ATB group contained *Enterobacteriaceae*, *Corynebacteriales*, and *Gammaproteobacteria* (Xu et al., 2022) (**Figures 1C**, **2**). ATB can deplete bacterial colonies that are propionogenic, having the potential to produce short-chain fatty acids (SCFAs) essential for T-cell function, which are necessary for efficacy of ICIs and control of tumorgenesis (DiPalma and Blattman, 2023).

Microbiota diversity in patients significantly influences the immune response during tumorigenesis; individuals undergoing anticancer treatments have demonstrated a strong correlation between specific commensal bacteria and enhanced protective antitumor T-cell responses. Patients receiving PD-L1 inhibition therapies showed improved treatment efficacy when *Bifidobacteria* species were present in their microbiome (Gopalakrishnan et al., 2018). Patients treated with vancomycin exhibited more effective radiotherapy outcomes on tumor lesions when levels of immunosuppressive metabolites, specifically butyrate and propionate, derived from *Clostridiales*, were lowered post-antibiotic treatment (Sepich-Poore et al., 2021). This suggests that the concentration of propiongeneic species in the microbiome influences treatment efficacy. Lower abundances may impair immune cell function and contribute to treatment resistance. Since many therapies depend on functional T-cells, their dysregulation by microbiome shifts caused by ATB could directly undermine therapeutic outcomes.

It may seem counterintuitive that antibiotics are associated with a reduced efficacy of ICI in cancer therapy, given that decreases in microbial diversity are associated with increases in immune activation (Gopalakrishnan et al., 2018; Wilson et al., 2020). This paradox is likely explained by the loss of specific bacterial species, such as *A. muciniphila* and *Ruminococcaceae*, that are associated with improved ICI responses. Antibiotics may specifically reduce these “favorable” microbial species. Supporting this, the restoration of *A. muciniphila* to the microbiome can reinstate the efficacy of PD-1 blockade in a T cell-dependent manner in a mouse tumor model (Mager et al., 2020; Wang et al., 2024). Second, much like autoimmunity and HIV/AIDS progression, cancer is a long-term disease, and the rules for how alterations to the microbiome in acute versus chronic disease situations may be quite different. Decreased microbial diversity is associated with faster disease progression rather than improved prognosis. One may infer then that decreased microbial diversity and increases in circulating microbial products/metabolites may provide an advantage to the tumor rather than to the immune system.

## Transplant and immunosuppressed patients in the ICU

Microbiome health is also critical in solid organ transplant recipients (SOTR), who face a heightened risk of severe infections such as *C. difficile* infection (CDI) and recurrent CDI (rCDI), both linked to higher mortality, especially in liver transplant recipients (Rodig et al., 2023; Almohaya et al., 2024) (**Figure 2**). Studies in lung, kidney, and liver transplant patients have further shown that CDI and multidrug-resistant bacteria (MDRB) contribute to increased mortality (Chan et al., 2020; Ponholzer et al., 2024) (**Figure 2**). Often, these infections are treated with antibiotics and/or fecal transplants, which have been shown to restore the GI microbiome to a healthy state, implying a link between severe infection in SOTRs and their microbiome health. Further, SOTRs with gut dysbiosis are at an increased mortality risk (Swarte et al., 2022). As SOTR are already at increased risk of hospitalization, and individuals hospitalized in the ICU setting have a significantly increased dysbiosis due to various factors, understanding the relationship between microbiome health and SOTR mortality is principal (Donnelly et al., 2015; Szychowiak et al., 2022).

One study showed loss of microbial diversity in liver transplant patients was associated with acute cellular rejection (ACR) and bloodstream infections (BSI). In ACR, *Bacteroides, Enterobacteriaceae, Streptococcaceae*, and *Bifidobacteriaceae* were increased, whereas *Enterococcaceae, Lactobacillaceae*, *Clostridiaceae*, *Ruminococcaceae*, and *Peptostreptococcaceae* were decreased (Kato et al., 2017) (**Figure 2**). Within the Diaz et al. study evaluating the salivary microbiome of patients who underwent kidney and heart transplants, their microbiome was disrupted by opportunistic pathogenic species, including *Enterobacteriaceae, Pseudomonas* and *Acinetobacter* (Diaz et al., 2013). Certain species from the following families can be considered pathogenic *Bacteroides, Pseudomonas, Acinetobacter Enterobacteriaceae, Streptococcaceae*, and *Bifidobacteriaceae* (Kato et al., 2017). Even though some species of Bacteroides are among the dominant beneficial gut microbes, there are species that are considered pathogenic (Brown et al., 2019; Zafar and Saier, 2021). Commensal bacteria provide nutrients, reduce opportunistic microbes, assist in digestion, and modulate the immune system. Surgery, antibiotics, immunosuppressants, and other treatments can disrupt the microbiome in which pathogenic bacteria outcompete the commensal bacteria. Some of these opportunistic microbes, such as those within the *Enterobacteriaceae* family, can release immunogenic substances such as endotoxin lipopolysaccharide (LPS), which can induce an inflammatory response. Additionally, there is emerging evidence that has shown a relationship between the gut microbiome and solid organ transplant-associated pathogenic infections. One of the driving forces is associated with the interactions between the gut microbes and the host’s immune system. Studies have shown that the microbiota can influence innate and adaptive immune responses. Multiple studies using mouse models found that broad-spectrum antibiotics worsened outcomes and reduced the effectiveness of cancer immunotherapy compared to the control group (Nelson et al., 2015; Zou et al., 2021; DiPalma and Blattman, 2023). The exact mechanism affecting the immune system via the elimination of the microbiome is still unknown. Many studies suggest that it’s due to microbial-derived metabolites, such as short-chain fatty acids (SCFAs), that can influence the immune system (Gonçalves et al., 2018; DiPalma and Blattman, 2023). Additionally, other studies have shown that the microbiome can modulate T-cell homing (Yao et al., 2022). The reduction of beneficial microbes and increase in pathogenic microbes could potentially result in reduced T-cell homing, especially to mucosal sites (Yao et al., 2022). Overall, a healthy microbiome can reduce the number of infections in liver transplant recipients, a trend that may extend to other solid organ types (Chan et al., 2020).

In the context and heart transplantation, patients face a heightened risk of CDI and other nosocomial infections compared to recipients of other solid organs, highlighting a critical gap in our understanding (Donnelly et al., 2015). One potential clue to this increased risk of CDI is that in heart transplant recipients, immunosuppressive regimens have been associated with overgrowth of pathogenic microbial strains (Olek et al., 2023). The presence of pathogenic microbial strains in the mucosa could contribute to the etiology of harmful infections (Husebye, 2005). Shifts in microbiome composition in patients post-transplant and potentially in response to immunosuppressive therapy could result in other complications for patients. Lung transplant patients have been found to display a dysbiotic lung bacterial microbiome post-transplant and a higher chance of developing chronic lung allograft dysfunction (CLAD), which is characterized by hyperimmune activation in lung and airway tissues as well as an increased risk of allograft rejection (McGinniss et al., 2021; Wu et al., 2023).

The top five causes of death in kidney transplant recipients with allograft function is infectious complications (Chan et al., 2020) Several studies have shown a relationship between the pathogenesis of transplant associated infection due to the disruption of the microbiome post kidney transplant (Chan et al., 2020). Diarrhea is a common complication post kidney transplantation, which is thought to be due to the immunosuppressant mycophe-nolate mofetil (MMF). One study showed that within a cohort of 97 kidney transplant patients, 40 individuals who experienced post-transplant diarrhea had a significantly lower median Shannon diversity within the fecal specimens than the non-diarrhea group (Zhang et al., 2021). Additionally, *Ruminococcus*, *Bacteroides Dorea*, and *Coprococcus* were also significantly lower in the diarrhea group (Zhang et al., 2021). The aforementioned groups of genus contain species that are considered beneficial commensal bacteria within the gut, with *Bacteroides* being one of the most abundant within the gut.

In the case of hematopoietic stem cell transplants (HCSTs), those who receive allogeneic HCSTs with more diverse gut microbiomes have lower HCSTs-related mortality and increased overall survival compared with patients with lower microbial diversity (Gopalakrishnan et al., 2018). These studies suggest that greater microbial diversity is often associated with positive transplant patient outcomes and that more research is needed to determine if similar microbiome-driven survival benefits extend to lung and heart transplant recipients.

Differences in immunosuppression dosage across different transplant types may impact disparities of infection risk (**Figure 1D**). For example, maintenance dosing of tacrolimus, a commonly used immunosuppressant, in renal transplant recipients are typically 6–10 ng/mL trough levels, while cardiac transplant recipients are 10–15 ng/mL trough levels (Arnol et al., 2020; Lindenfeld et al., 2004).

A study using a mouse model, evaluated the effects of tacrolimus treatment which resulted in a significant shift in the abundance of *Bacteroides*, *Allobaculum* (P <.01), and *Lactobacillus* (P <.05), a decrease in *Clostridium* (P <.01), *Ruminococcus*, *Rikenella*, *Ruminococcaceae* (P <.05) as well as an increase in CD4 +CD25 hiFoxP3 + regulatory T cells in the blood and mucosa (Zhang et al., 2018). The significant difference was observed in both high dose tacrolimus treated and treatment via fecal transplant from a high dose tacrolimus-treated donor (Zhang et al., 2018). There was no significant change in the microbiota within the low dose (0.1 mg/kg) of tacromilus. Within a human study evaluating the impact of immunosuppressive treatments, everolimus plus mycophenolate mofetil (n = 9) vs tacrolimus plus mycophenolate mofetil (n = 11), on the gut microbiota within renal transplant patients showed no significant difference on the taxonomic level between the two groups but showed a difference in functional genes (Zaza et al., 2017). Within the Zaza et al. study, they did not have a control group without immunosuppressant treatment to compare to. Although there was no difference between the two groups treated with immunosuppressants, there may be a microbiome difference between patients receiving or not receiving immunosuppressants. Outside of the groups listed above, there is still a wide variety of patients with dysregulation of their immune systems from autoimmune diseases and treatments for these diseases. In mouse models, mice susceptible to developing rheumatoid arthritis (RA) showed a significant decrease in microbial diversity, even before disease onset. Within a human fecal genomic sequencing study, patients with RA were found to have increased concentration of pathogenic microbes and a concurrent decrease in commensal organisms (Lindenfeld et al., 2004). Current research indicates these changes increase the immune system’s inflammatory responses, which can, therefore, degrade intestinal immune barriers and predispose a patient who is already prone to developing RA to develop more severe disease manifestations.

The causality of changes in the microbiome is less certain. Microbiomes of patients with Inflammatory Bowel Disease (IBD) show decreased diversity, including between areas of inflammation and without inflammation within the digestive tract (Shan et al., 2022). One mechanism by which this dysbiosis may be associated with the worsening of IBD presentation is that it can result in changes in the glycosylation of intestinal barrier cells, resulting in damage to the junctional proteins and upregulation of pro-inflammation gene transcription (Macfarlane et al., 2009).

## Modulating the microbiome to alter health outcomes in the ICU

Another possibility was set forth by *Brenchley* et al (Brenchley et al., 2006), in which circulating microbial products are correlated with systemic immune activation in HIV patients and more rapid progression to AIDS. It may be inferred, then, that the microbiome may have systemic effects in another setting of immune activation and suggests that differences in the microbiome can impact immune function in the settings of transplant, autoimmunity, and responsiveness to immune checkpoint blockade for cancer. Research that has uncovered how the microbiome’s composition can impact patients’ outcomes in the ICU has also implied that alterations to it may improve treatment outcomes. Here, we describe potential modifications that may alter health outcomes and improve patient prognosis in ICU patients.

### General patients in the ICU

One practical approach to ensuring positive outcomes for ICU patients is medical nutrition therapy (MNT), which involves nutritional interventions to manage critical conditions. For instance, a recent study demonstrated that administering a fiber-based diet in ICU patients receiving ATB allowed for colonizing bacteria that metabolize fiber into SCFAs associated with resistance to MDRB (Freedberg et al., 2020). However, the administration of MNT is not without risk; interventions must be approached with caution in critically ill patients, as impaired gut motility and barrier dysfunction could increase the risk of adverse events. More recent studies seek to characterize specific nutritional components that beneficially modulate the microbiome while minimizing possible adverse events, though these findings have yet to be published.

### Cancer patients in the ICU

While the relationship between the microbiome and cancer progression is complex, much research suggests that its modulation can improve therapy outcomes. Promising interventions include probiotics, prebiotics, synbiotics, nutrition, and fecal microbiota transplant (FMT). Given the growing interest in these interventions, their potential application in critically ill cancer patients, including those requiring mechanical ventilation, has also gained attention.

In some cases, mechanical ventilation can lead to ventilator-associated pneumonia (VAP) within 48 hours, which is considered the most common complication in critically ill patients. The impact of probiotics and synbiotics on critically ill patients undergoing mechanical ventilation remains unclear. A meta-analysis evaluating 30 randomized clinical trials, where patients were administered with probiotics or synbiotics, suggested that within critically ill patients, probiotic intervention decreased the rate of infection by 20% and the rate of VAP by 25-30% (Manzanares et al., 2016) (**Table 1**). Additionally, within a randomized controlled trial (n = 72), evaluating the impact of synbiotics in reducing complications in mechanically ventilated patients with sepsis, the results indicated that the incidence of enteritis and VAP were significantly (p < 0.05) lower in patients administered with synbiotics (Shimizu et al., 2018) (**Table 1**). This study showed a significant increase in the total bacterial number of *Lactobacillus* and *Bifidobacterium* within the gut microbiome of the synbiotics group than the non-synbiotics group (Shimizu et al., 2018) (**Table 1**). Other factors, such as mortality rate and number of ventilator-free days at 28 days showed no statistical significance between the groups (Shimizu et al., 2018) (**Table 1**). In contrary, a randomized, double-blind study (n = 259) evaluating the impact of Synbiotic 2000 FORTE^®^ on VAP and mortality within mechanically ventilated ICU patients showed no significant difference in the incidence of VAP or mortality between the synbiotic and non-synbiotic groups (Knight et al., 2009) (**Table 1**). Note that the *Shimizu* et al. study evaluated mechanically ventilated patients with sepsis; meanwhile, the Knight et al. did not. An additional study by *Saikrishna* et al., evaluating the effects of probiotics on ICU patients (n = 35) and the prevalence of VAP and ventilator-free days also found no statistical difference between probiotic and non-probiotic groups (Saikrishna et al., 2023) (**Table 1**). Even though the aforementioned study showed a statistical difference in microbiome composition between probiotic and non-probiotic groups, including the presence of *Lactobacillus paracasei, Lactobacillus rhamnosus*, and *Streptococcus thermophilus* (Saikrishna et al., 2023), variations in these results could stem from sample size, duration, and type of probiotics/synbiotics, as well as a single-center study vs multi-center study and variation in ICU patient diagnosis, to name a few (**Table 1**). Within these studies, patients with cancer are included if they meet the study criteria, but they are not the sole study population. Further research is required to investigate the conditions in which probiotics and/or synbiotics are clinically effective for ICU patients. Understanding the conditions would further our knowledge of why studies have varying results and what factors maximize the benefits. Regardless once we further our understanding probiotics and synbiotics should be considered for clinical use, but this would require FDA approval and further research. Other than probiotics and synbiotic as a therapeutic option, nutrition and diet can be of therapeutic value since diet can modify the microbiome. The benefit of leveraging nutrition and diet is that this does not require FDA approval. Regardless, this would require further research to understand what types of nutrition and diet would benefit patients in the ICU.

**Table 1:** Study comparisons of ICU patients administered with probiotics or synbiotics.

Reference	Sample Size	Type of Probiotics or Synbiotics	Clinical Findings
Saikrishna et al.	n = 35	Probiotics: VSL#3^®^ Capsule	No significant difference in prevalence of VAP and ventilator-free days between probiotic and non-probiotic groups. Statistical difference in microbiome composition between probiotic and non-probiotic groups, including the presence of *Lactobacillus paracasei, Lactobacillus rhamnosus*, and *Streptococcus thermophilus*
Knight et al.	n = 259	Synbiotics: Synbiotic 2000 FORTE^®^	No significant difference in the incidence of VAP or mortality between the synbiotic and non-synbiotic groups.
Shimizu et al.	n = 72	Synbiotics: *Bifidobacterium breve* strain *Yakult*, *Lactobacillus casei* strain *Shirota*, and galactooligosaccharides	Incidence of enteritis and VAP were significantly (p < 0.05) lower in patients administered with synbiotics. Significant increase in the total bacterial number of Lactobacillus and Bifidobacterium within the gut microbiome of the synbiotics group. Mortality rate and number of ventilator-free days at 28 days showed no statistical significance.
Manzanares et al.	Meta-analysis of 30 randomized clinical trials	Probiotics: *Lactobacillus plantarum*, *Lactobacillus rhamnosus* strain GG	Probiotic intervention decreased the rate of infection by 20% and the rate of VAP by 25-30%

Patients with cancer are prone to infection, leading to their admittance to the ICU and administration of antibiotics. A significant concern is MDRB linked to high mortality rates, such as carbapenemase-producing enterobacteria (CPE), extended-spectrum beta-lactamase-carrying strains, and vancomycin-resistant *Enterococcus* (VRE). As previously mentioned, cancer studies have indicated that antibiotic exposure can lead to dysbiosis and reduced treatment efficacy such as ICIs (Tsikala-Vafea et al., 2021). The effects of ATB can lead to dysbiosis, which can introduce pathogenic bacteria. Several proposed interventions include supplementing with probiotics/synbiotics, fecal transplants, and diet. Within a randomized double-blind, placebo-controlled trial (n = 120), patients were treated with amoxicillin-clavulanate antibiotics for 10 days and compared the effects of a 30-day intervention with placebo *Saccharomyces boulardii* CNCM I-745^®^ and a probiotic treated group enriched with *Lactobacillus paracasei* Lpc-37, *Lactobacillus acidophilus* NCFM*, Bifidobacterium lactis* Bl-04*, Saccharomyces boulardii*, and *Bifidobacterium lactis* Bi-07 (Bactiol duo^®^) (Wieërs et al., 2021). The results showed a significant decrease in *Pseudomonas* after treatment with probiotics post-antibiotic treatment (P < 0.05) (Wieërs et al., 2021). Even though there was a transient increase in AmpC-producing enterobacteria after antibiotic treatment, the probiotic group had a significant decline (P<0.05) compared to the placebo group (Wieërs et al., 2021). The overall study claims an association of *Saccharomyces boulardii* paired with specific *Lactobacillus* and *Bifidobacterium* species decreasing the number of antibiotic-resistant pathogens within the gut and thus impacting antibiotic treatment (Wieërs et al., 2021).

### Transplant and immunosuppressed patients

Noting that decreases in microbial diversity are associated with more severe impacts on transplant patients, several potential approaches to increasing gut microbiome diversity in patients have been investigated (Chan et al., 2020). These include prebiotics (non-digestible food ingredients), probiotics (often bacteria and yeast-supplemented to increase microbial diversity), and nutrition-based interventions. While prebiotics have not been extensively studied for SOTR patients, there is evidence that hospitalization, especially in the ICU, can significantly alter dietary intake and lead to dysbiosis. Initial studies into SOTR probiotics have begun, focusing on liver transplant patients (Chan et al., 2020). Further, some studies suggest that critically ill patients who were given enteral nutrition with a high-protein diet enriched with arginine, fiber, and antioxidants had a significantly lower catheter-related sepsis rate than patients fed a standard high-protein diet. However, it is important to note that the use of microbiome-targeted interventions in SOTR patients remains limited by a lack of large-scale clinical trials. Resultantly, concerns about probiotic safety and variability in patient response still need to be further elucidated.

Beyond SOTRs, microbiome-related immune modulation has been implicated in other disease settings, including autoimmunity, HIV, and hematopoietic stem cell transplant (HSCT). In HSCT, in which patients that develop graft-versus-host disease (GVHD) have a higher propensity for gut microbiota dysbiosis (Bhatia et al., 2007; Taur et al., 2014). Notably, this dysbiosis mainly manifests as decreased microbial diversity, resulting in changes in microbial composition and differences in microbiome-derived metabolites, consistent with the model proposed by Brenchley et al. (2006) in HIV patients, may result in global increases in inflammation and immune activation resulting in predictors of poorer prognosis in GVHD patients. AS such, microbiome modulation strategies, such as fecal transplant or other methods described above, could significantly decrease GVHD in HSCT patients by concomitant decreases in inflammation and immune function.

Additional studies have shown that probiotics can reduce infection rates in liver transplant recipients (Zhang et al., 2013; Lederer et al., 2017). This indicates that including probiotics in the treatment regimen of SOTR and other immunocompromised individuals within the ICU setting may decrease infection rates. However, these solutions may be hindered by the lack of FDA-approved probiotics, creating a barrier to administer in a hospital or ICU setting as doctors cannot prescribe specific probiotics. Lack of regulation of probiotics may increase the risk of introducing unhealthy bacteria to patients due to contamination. On the other hand, prebiotics may be easily included by modifying the patient’s diet under the guidance of a nutritionist. As our understanding of the microbiome expands, so must the treatment options for clinicians, including probiotics that may be prescribed, so that they can harness this expanded understanding for improved patient outcomes.

## Discussion

Further research into how modulations of microbiome composition affect immunocompromised patients is essential for advancing current treatment modalities. Microbial imbalances can lead to adverse health outcomes, particularly in vulnerable populations such as ICU patients. Often, dysbiosis in these patients can be exacerbated by HAIs, among other factors. Studies have shown that microbial dysbiosis can result not only in complications such as infection, organ failure, and increased mortality in ICU patients but can be even more devastating for those with compromised immune systems. This can lead to increased toxicity, decreased effectiveness of anticancer agents, and adverse health outcomes in patients undergoing cellular therapy treatments (Gopalakrishnan et al., 2018; Sepich-Poore et al., 2021). Thus, studies that aim to reduce or reverse this dysbiosis need to be better understood to improve ICU patients’ prognosis.

One area of research that holds the potential to skew dysbiosis in ICU patients is oral supplements that can be administered to patients before or during hospital admission to alter their microbiome and later bias those patients toward positive outcomes. For instance, using probiotics alone or combined with prebiotics in ICU patients prevented them from developing ventilator-associated pneumonia (VAP) (Batra et al., 2020). The use of prebiotics in the ICU to alter the microbiome’s composition in patients holds much promise, as adding prebiotics or alternative nutrition that contains prebiotics can be easily changed and does not require FDA approval. Recent investigations have focused on nutrition’s impact on modulating the gut microbiome’s composition (Cresci, 2025). Some recent studies have indicated that applying fiber-rich diets increased the abundance of *Bifidobacterium* species, which are often involved in cross-feeding with other gut microbes (Oliver et al., 2021). Additionally, low-fiber diets promote the expansion of mucosa-degrading bacteria, while high-fiber diets can restore healthier microbiome composition (Desai et al., 2016). These findings suggest that targeted nutritional strategies could be an easy way to tailor the microbiome to shape the microbiome toward a more beneficial state.

Subsequent studies analyzed the impact of fecal microbiota transplantation on patients in the ICU with confirmed dysbiosis, demonstrating the reversal of dysbiosis post-transplant (Alagna et al., 2019). However, the lack of FDA regulation currently limits oral therapies that could potentially skew microbiome composition and prevent or reverse dysbiosis. They require more development before they can be applied clinically. It is also essential to consider the potential negative impacts of probiotics or microbiome transplantation before their administration to patients, as potential adverse effects can occur (Dailey et al., 2019). For instance, it is possible that gut flora associated with the pathobionts could be introduced into a patient’s microbiome, especially post-fecal transplantation, potentially leading to adverse health outcomes and unintentional infections, such as the development of antibiotic-resistant bacteria, which has been seen in some patient cases post-fecal transplant (CH and CT, 2020). Additionally, standard methods of fecal microbiota transplant administration, such as colonoscopy or nasogastric tube delivery, can result in psychological stress to patients, causing them to meet with hesitation or resistance (Qu et al., 2022). Further, differences in donor-related variability could result in inconsistent outcomes, as there is currently no consensus on what an “ideal” donor looks like. All these potential contradictions should be considered when deciding to administer FMT.

Another promising research avenue is gaining a deeper understanding of the pathobiome, which, if understood, could help us design treatments that eliminate pathogenic microbes and skew patient responses in a more favorable direction. When colonized with microbes that drive inflammatory responses, patients have been found to have an increased risk of complications associated with inflammation, including sepsis (Alverdy and Krezalek, 2017). Targeting specific inflammatory microbes in the pathobiome could be a potential intervention with the ICU, but more research is required to understand this complex dynamic. The interplay between the microbiome shift to a pathobiome, the gut environment, microbial byproducts, and the immune system is not well understood. Most current research investigates the reestablishment of a healthy microbiome via probiotics, synbiotics, nutrition, antibiotics, fecal microbiota transplant, and immune-boosting strategies, but there is a lack of research focusing on a therapeutic approach to targeting specifically the inflammation-causing pathobiome. Further studies that aim to identify which species are involved in negative or positive patient outcomes and learn which microbial species promote health may also be beneficial in developing future therapeutic interventions. Ultimately, they hold the potential to exploit these findings to improve patient outcomes in the ICU.
